# Valence atom with bohmian quantum potential: the golden ratio approach

**DOI:** 10.1186/1752-153X-6-135

**Published:** 2012-11-12

**Authors:** Mihai V Putz

**Affiliations:** 1 Laboratory of Computational and Structural Physical Chemistry, Biology-Chemistry Department, West University of Timişoara, Pestalozzi Street No.16, Timişoara, RO-300115, Romania

**Keywords:** Electronegativity, Chemical hardness, Bohmian mechanics, Heisenberg imbalance equation, Slater electronic density

## Abstract

**Background:**

The alternative quantum mechanical description of total energy given by Bohmian theory was merged with the concept of the golden ratio and its appearance as the Heisenberg imbalance to provide a new density-based description of the valence atomic state and reactivity charge with the aim of clarifying their features with respect to the so-called DFT ground state and critical charge, respectively.

**Results:**

The results, based on the so-called double variational algorithm for chemical spaces of reactivity, are fundamental and, among other issues regarding chemical bonding, solve the existing paradox of using a cubic parabola to describe a quadratic charge dependency.

**Conclusions:**

Overall, the paper provides a qualitative-quantitative explanation of chemical reactivity based on more than half of an electronic pair in bonding, and provide new, more realistic values for the so-called “universal” electronegativity and chemical hardness of atomic systems engaged in reactivity (analogous to the atoms-in-molecules framework).

## Introduction

Recently, the crucial problem regarding whether chemical phenomena are reducible to physical ones has had an increasingly strong impact on the current course of conceptual and theoretical chemistry. For instance, the fact that elements arrange themselves in atomic number (Z) triads in approximately 50% of the periodic system seems to escape custom ordering quantifications [1,2]. The same applies to the following: the fascinating golden ratio (*τ*) limit for the periodicity of nuclei beyond any physical first-principle constants, which provides specific periodic laws for the chemical realm [3-6]; the fact that atoms have no definite atomic radii in the sense of a quantum operator, and even the *Aufbau* principle, which, although chemically workable, seems to violate the Pauli Exclusion Principle [7]; at the molecular level, the well-celebrated reaction coordinate, which, although formally defined in the projective energy space, does not constitute a variable to drive optimization in the course of chemical reactions, appearing merely as a consequence of such reactions [8]; the problem of atoms in molecules [9], i.e., how much of the free atoms enter molecules and how much independency the atoms preserve in bonding; and chemical bonding itself, which ultimately appears to be reinterpreted as a special case of bosonic condensation with the aid of bondons – the quantum bosons of chemical bonding, which, without being elementary, imbue chemical compounds with a specific reality [10,11].

In the same context, the specific measure of chemical reactivity, electronegativity (χ), which lacks a definite quantum operator but retains an observable character through its formal identity with the macroscopic chemical potential *χ=-μ*[12,13], was tasked with carrying quantum information within the entanglement environment of Bohmian mechanics [14-17] and has thus far been identified with the square root of the so-called quantum potential *χ* = *V*_*Q*_^1/2^[6].

However, the striking difference between an atom as a physical entity, with an equal number of electrons and protons (thus in equilibrium), and the same atom as a chemical object, with incomplete occupancy in its periphery quantum shells (thus attaining equilibrium by changing accepting or releasing electrons), is closely related to the electronegativity phenomenology in modeling chemical reactivity. Moreover, this difference triggers perhaps the most important debate in conceptual chemistry: the ground vs. valence state definition of an atom.

**Figure 1:**
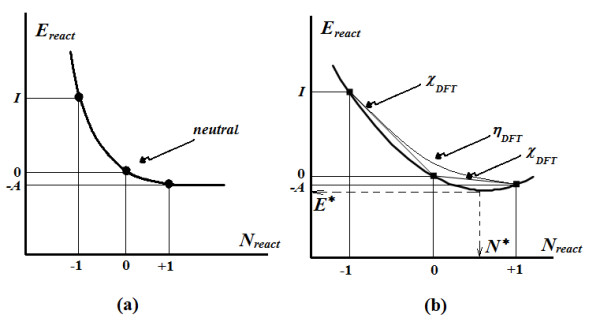
**The two energy curves (thick lines) for the quantum atom in (a) the apparent or *reactive ground state *and (b) the shifted or *critical ground state.***

The difficulty may be immediately revealed by considering the variation in the total energy (of the ground and/or valence state – see below for an explanation of their difference) around the physical equilibrium (neutral atom) attained between the release (by ionization, *I*) and receipt (through affinity, *A*) of electrons toward chemical equilibrium (in molecules, chemical bonding). Accordingly, the curve passing through these points apparently only behaves as shown in Figure 1(a), while in all systems (with numerical *I* and *A*), the obtained interpolating curve presents a minimum toward accepting electrons (see Figure 1(b)), thus confirming the electronegativity concept as a chemical reality, although with a predicted fractional charge (for example, the critical charge N^*^) on an atom at chemical equilibrium (i.e., not reducible/comprehensible to/by an ordinary physical description of atoms).

However, the physical-to-chemical paradox continues in an even more exciting fashion as follows. When, in light of the above discussion, electronegativity is recognized with the two-point limits shown in Figure 1(b), namely [13,18]

(1) $$\chi =\{\begin{array}{l} I\;\ldots N_{\mathrm{react}}\in \left[-1,0\right)\\ A\ldots N_{\mathrm{react}}\in \left(0,+1\right] \end{array}$$

the limits represent tangents to a curve that does not describe chemical equilibrium but an excited state driven by the parabolic form

(2) $$E_{\mathrm{DFT}}=-\chi N+\eta N^{2}$$

which happens to correspond to the celebrated density functional theory (DFT) working energy expression [13,19-21] written in terms of electronegativity and chemical hardness, respectively defined as follows [13,22-25]:

(3) $$\chi_{\mathrm{DFT}}=-{\left(\frac{\partial E}{\partial N}\right)}_{V\left(r\right)}$$

(4) $$\eta_{\mathrm{DFT}}=-\frac{1}{2}{\left(\frac{\partial \chi }{\partial N}\right)}_{V\left(r\right)}=\frac{1}{2}{\left(\frac{\partial^{2}E}{\partial N^{2}}\right)}_{V\left(r\right)}$$

The point is that curve (2) is not chemically minimized, although it is very often assumed to be in the DFT invoked by the chemical reactivity literature [13,26-29]; however, the curve cannot be considered indicative of a sort of ground state (neither reactive nor critical states of Figure 1). Additionally, by comparing the curves of Figure 1 (a) and (b), the curve of eq. (2) occurs above both the reactive and critical curves of Figure 1; it thus should represent the *chemical valence state* with which to operate. Therefore, much caution should be taken when working with eq. (2) in assessing the properties of atoms, molecules, atoms in molecules, etc. Nevertheless, this is another case of chemistry not being reducible to physics and should be treated accordingly. It is worth noting that Parr, the “father” of eq. (2) and a true pioneer of conceptual density functional theory [30,31], had tried to solve this dichotomy by taking the “valence as the ground state of an atom in a perturbed environment”. This statement is not entirely valid because perturbation is not variation such that it may be corrected by applying the variational principle to eq. (2), for example. In fact, using such variation should be considered a double variational technique that is necessary to arrive at the celebrated chemical reactivity principles of electronegativity and chemical hardness, as recently shown [32].

The current line of work takes a step forward by employing the double variation of the parabolic energy curve of type (2) to provide the *quantum* (DFT) *valence charge* of an atom (say, N^**^) and to compare it either quantitatively and qualitatively with the chemical critical charge N^*^. The goal of these efforts is to gain new insight into the valence state and chemical reactivity at the quantum level. To this end, the relation of Bohmian mechanics to the concept of the golden ratio will be essential and will be introduced in the following.

The consequences of the joint consideration of Bohmian mechanics and the golden ratio for the main atomic systems will be explored, and the *quantum chemical valence state* will be accordingly described alongside the so-called universal electronegativity and chemical hardness, refining the work of Parr and Bartolotti [33] as well as generalizing the previous Bohmian-Boeyens approach [3,4].

## Background methods

Two apparently disjoint theories of matter will be employed to characterize the quantum valence of an atom: *Bohmian mechanics* – furnishing the main equation for total energy – and the *fundamental quantum mechanics* through the Heisenberg combined with de Broglie principles providing the wave-particle indeterminacy framework in which the golden ration dependency of *Z/N* naturally appears as quantifying the valence states of atoms considered the “ground state” of the atomic chemical reactivity.

### Bohmian mechanics

Because of the need to reduce Copenhagen’s indeterminacy for quantum phenomena, i.e., by associating it the quantum description of “Newtonian” forms of motion, though by preserving probability densities, quantum averages, etc., the so-called “minimalist” quantum theory may be formulated following the Bohm quantum mechanical program as follows.

One begins with the general eikonal wave-function form [14]

(5) $$\psi \left(r,t\right)=R\left(r\right)\exp\left(\frac{i}{\hbar }S\left(r,t\right)\right)$$

which represents the mid-way between wave and particle mechanics because it contains both information regarding Hamilton-Jacobi theory and the Wentzel-Kramers-Brillouin (WKB) approximation [34] through the principal phase function *S*(*r,t*) while preserving the amplitude relationship with the systems’ quantum density:

(6) $$\rho \left(r\right)=\psi^{2}\left(r\right)=R^{2}\left(r\right)$$

In this framework, the Schrödinger equation,

(7) $$i\hbar \frac{\partial }{\partial t}\psi \left(r,t\right)=-\frac{\hbar^{2}}{2m}\nabla_{r}^{2}\psi \left(r,t\right)+V\left(r\right)\psi \left(r,t\right)$$

decomposes into real and imaginary parts. The real part can be expressed as follows:

(8) $$\frac{\partial S\left(r,t\right)}{\partial t}+\frac{{\left(\nabla_{r}S\left(r,t\right)\right)}^{2}}{2m}-\frac{\hbar^{2}}{2m}\frac{\nabla_{r}^{2}R\left(r\right)}{R\left(r\right)}+V\left(r\right)=0$$

representing a continuous “fluid” of particles driven by the “guidance” momentum:

(9) $$mv=p=\nabla_{r}S\left(r,t\right)$$

moving under a joint external potential *V*(*r*) as well as under the so-called quantum potential influence:

(10) $$V_{Q}\left(r\right)=-\frac{\hbar^{2}}{2m}\frac{\nabla_{r}^{2}R\left(r\right)}{R\left(r\right)}$$

The consequences are nevertheless huge. For example, this methodology allows for the interpretation of the trajectories orthogonal to constant surfaces, by cancelling the Laplacian of the wave fronts ∇ _*r*_^2^*S*(*r*, *t*) = 0, which are obtained from eqs. (8) and (9) as the quantum equation of motion:

(11) $$\frac{\partial p}{\partial t}=-\nabla_{r}\left[V_{Q}\left(r\right)+V\left(r\right)\right]$$

Equation (11) resembles the classical Newtonian acceleration-force relationship only in a formal way; in fact, it generalizes it: it prescribes acceleration motion even in the absence of an external classical potential. This is essential in explaining why the inter-quark forces increase with the increase in inter-quark distances, no matter how great a separation is considered (a specific quantum effect), due to the presence of a quantum potential that does not fall off with distance as *V* does. It also nicely explains the observed interference patterns in double-slit experiments in the absence of classical forces. Alike, eq. (11) also appears suited for modeling chemical reactivity for the *valence atoms as free particles* in a virtually infinite potential environment to characterize their reactive behavior. In this regard, it is worth considering for such atoms the uniform motion by having ∂ *p*/∂ *t* = 0 through the time-constant associated wavefront condition and action *S*(*r=cnst.,t*)=*cnst*. (equivalent with Lagrangean constancy), in all given chemical space-points (atomic basins within molecule complex) [35]. This picture is also equivalently to have

(12) $$\frac{\partial S\left(r,t\right)}{\partial t}=0$$

applied to eq. (8). By doing so, one obtains

(13) $$\frac{{\left(\nabla_{r}S\left(r,t\right)\right)}^{2}}{2m}=-\left[-\frac{\hbar^{2}}{2m}\frac{\nabla_{r}^{2}R\left(r\right)}{R\left(r\right)}+V\left(r\right)\right]$$

which can be rearranged as follows:

(14) $$T=-V_{Q}-V\left(r\right)$$

such that the total energy of a the valence system is now entirely driven by the quantum potential:

(15) $$E_{Q}=T+V\left(r\right)=-V_{Q}$$

At this point, one can see that when turning to electronegativity and combining eq. (15) with DFT definition (3), one obtains a generalization of the previous Boeyens formulation [6]:

(16) $$\chi_{Q-DFT}={\left(\frac{\partial V_{Q}}{\partial N}\right)}_{V\left(r\right)}$$

which is the variation in the quantum potential with electron exchange under a constant classical or external potential.

However, for a quantum characterization of the valence state, we are interested in how the energy described by eq. (15) varies under a quantum potential (10)

(17) $$\begin{array}{l} E_{Q}(a.u.)=\frac{\nabla_{r}^{2}R\left(r\right)}{2R\left(r\right)}=\frac{\nabla_{r}^{2}\rho^{1/2}\left(r\right)}{2\rho^{1/2}\left(r\right)}\\ \;=\frac{1}{4}\frac{\nabla_{r}^{2}\rho \left(r\right)}{\rho \left(r\right)}-\frac{1}{8}\frac{{\left[\nabla_{r}\rho \left(r\right)\right]}^{2}}{\rho^{2}\left(r\right)} \end{array}$$

when the above relations (6) and (10) are substituted into eq. (15).

It is worth noting that although we obtained the total energy (17) in the Bohmian mechanics context, it showcases a clear electronic density dependency, not under a density functional (as DFT would require) but merely as a spatial function, which is a direct reflection of the entanglement behavior of Bohmian theory through the involvement of a quantum potential. However, in most cases, and especially for atomic systems, eq. (17) will yield numerical values under custom density function realizations.

### Golden ratio imbalance for valence states of atoms

Atomic stability and periodicity remain major issues in the structural theories of matter; fortunately, they both have been largely solved by wave-particle (W/P) complementarily quantum behavior; phenomenologically, such relationship can be expressed as “*WAVE* ⊗ *PARTICLE = constant*”, while it may be quantized (by Planck’s constant *h*) in the light of Heisenberg principle as [36]

(18a) $$\text{WAVE}⊗\text{PARTICLE}=n_{W/P}h$$

Remarkably, when fixing the particle’s observable property, say *O*, while letting wave information to vary, say Δ*O*, equation (18a) takes the workable form

(18b) $$\Delta O\times O=n_{O}h$$

having as the preeminent realization the Bohr-de Broglie formulation ^a^, leading with the first rationalization of the atomic periodicity [37]. However, when about the atomic chemical reactivity a similar analysis may be provided in terms of the number of electrons to atomic number ratio (*N/Z*): one may fix the observable (“particle”) character of the reactive atomic system by the ratio itself

(19a) $$O=\frac{N}{Z}$$

while modeling its evolving (“wave”) character by the natural variation of the previous ratio in terms of exchanged electrons respecting the neutral state:

(19b) $$\Delta O=\frac{\Delta N}{Z}=\frac{N-Z}{Z}$$

When combining eqs. (19a) and (19b) into eq. (18b) on the lowest quantized state (n_*O*_*=*1), the “ground state” of atomic reactivity that is the atom in its *valence* state so to speak, and within atomic units’ formulation (i.e. by putting *h*=1, since the actual reactivity quantification involves only numbers with no dimension), one has the so called *Heisenberg imbalance equation* for *valence atoms*

(20a) $$\frac{N-Z}{Z}\times \frac{N}{Z}=1$$

that can be rewritten as

(20b) $$Z^{2}+NZ-N^{2}=0$$

Eq. (20b) has the elementary acceptable solution

(21a) $$Z=\frac{-N+\sqrt{N^{2}+4N^{2}}}{2}=N\tau$$

which establishes, the direct “chemical” connection between the number of electrons and the atomic charge by means of the golden ratio

(21b) $$\tau =\frac{-1+\sqrt{5}}{2}=0.6180$$

generalizing the “physical” connection between nuclear (cosmic) synthesis at high pressure and atomic stability in the gas phase (*Z=N*); one has therefore the actual physical-to-chemical *electronic charge – atomic number* relationships

(22) $$\frac{Z}{N}=\{\begin{array}{l} \text{1…}STABLE(PHYSICAL)ATOM\\ \tau \ldots REACTIVE(CHEMICAL)ATOM \end{array}$$

Worth remarking the results of type (20) and (22), here based on chemical reactivity specialization of Heisenberg type equations (18a) and/or (18b), were previously obtained at the level of neutron-protonic imbalance, inside the atomic nuclei, based on well-founded empirical observations [6]. The present golden ratio appearance is ultimately sustained also by the deviation from the *N=Z* condition for so-called “quark atoms” (as another way in considering the atoms in a quantum valence state), earlier identified as true matter’s entities responsible for matter’s reactivity at the atomic level [38].

Therefore the atomic structure branching (22) can be regarded as the present *golden ratio* extension *to valence atom* and as such employed; actually, its consequences regarding the characterization of the quantum valence states of atoms within the Bohmian quantum potential are the main aims of the present endeavor and will be discussed next.

## Atomic implementation and discussion

### On Slater density for valence atoms

Density is considered a “goldmine” in current computational and conceptual quantum chemistry due to its link with observable quantities, energy density functionals in particular, as celebrated by DFT [13,20,39,40]. However, to quantitatively approach the chemical phenomenology presented in Figure 1, involving the ionization-to-affinity atomic description, the general Slater [33] density (involving the orbital parameter *ξ* dependency) will be here employed for the first trial on modeling the combined Bohmian and gold-ratio features of valence atom; it assumes the general (trough still crude) working form:

(23a) $$\rho \left(r,\xi \right)=\rho_{0}\exp\left[-2\xi \;r\right]$$

For the reactivity at the valence atomic level, or for some outer shell (*n*) considered at the atomic frontier, one may assume almost electronic free motion or at least electronic motion under almost vanishing nuclear potential *V*(*r*); this way the density (23a), while entering the quantum potential (10) recovers the negative kinetic energy by the virial identity (14). Analytically, since eqs. (6), (10) and (23a), one has ∇ _*r*_^2^*ρ*^1/2^ = *ξ*^2^*ρ*^1/2^ and the actual valence atomic virial realization looks like

(24) $$V_{Q}\left(r\right)=-\frac{\hbar^{2}\xi^{2}}{2m}\ldots =-T=-\frac{p^{2}}{2m}$$

Equation (24) leaves with the identity:

(25a) ℏξ=p

that may be further rewritten with the help of the atomic Bohr-de Broglie relationship (see the note ^a^) to provide the atomic frontier radii shell-dependency

(25b) $$r_{\mathrm{frontier}}=\frac{n}{\xi }$$

Remarkably, the same result is obtained when employing a far more reach atomic shell structure description, namely when starting with the full atomic radial Schrödinger density [25]

(26) $$\rho_{n}\left(r,\xi \right)=4\pi r^{2}\frac{{\left(2\xi \right)}^{2n+1}}{\left(2n\right)!}r^{2n-2}\exp\left[-2\xi \;r\right]$$

and imposing the null-gradient condition [41], ∇_*r*_*ρ*_*n*_(*r*, *ξ*) = 0, in accordance with the celebrated Bader condition of electronic flux of atoms-in-molecules [9,42], to yield:

(25c) $$r_{\max}=\frac{n}{\xi }$$

The identity between eqs. (25b) and (25c) gives sufficient support to the present Slater density approach eq. (23a) in modeling the valence atoms or the atoms at their frontiers approaching reactivity (i.e. atoms-in-molecules complexes by chemical reactions).

### Quantum chemical bonding and reactivity indices

Once convinced by the usefulness of the Slater density form (23a) for the present valence atomic analysis, one will next employ it under the so called Parr-Bartolotti form [33]

(23b) $$\rho \left(r,\xi \right)=N\frac{\xi^{3}}{\pi }\exp\left[-2\xi \;r\right]$$

such that to obey the *N*-normalization condition, as required by DFT [43-47],

(27) $$\int_{0}^{\infty }4\pi \;r^{2}\rho \left(r,\xi \right)dr=N$$

by applying the Slater integral recipe

(28) $$\int_{0}^{\infty }x^{n}\exp\left(-\alpha x\right)dx=\frac{n!}{\alpha {\;}^{n+1}}$$

It nevertheless showcases the parametric *ξ* dependency that can be smeared out by considering the variational procedure

(29) $$\frac{\partial E\left[\xi \right]}{\partial \xi }=0$$

upon applying the total atomic energy

(30) $$E\left[\xi \right]=T\left[\xi \right]+V_{\mathrm{ee}}\left[\xi \right]+V_{\mathrm{ne}}\left[\xi \right]$$

where the components are individually evaluated within a radial atomic framework with the respective results for [21,48]

● kinetic energy

(31a) $$\begin{array}{l} T\left[\xi \right]=\int_{0}^{\infty }4\pi \;r^{2}\;\left\{-\frac{1}{2}\left[\frac{1}{r^{2}}\frac{\partial }{\partial r}\left(r^{2}\frac{\partial }{\partial r}\right)\right]+\frac{\xi }{2r}\right\}\rho \left(r,\xi \right)dr\\ \;=N\frac{\xi^{2}}{2} \end{array}$$

● nucleus-electronic interaction

(31b) $$V_{\mathrm{ne}}\left[\xi \right]=-\int_{0}^{\infty }4\pi \;r^{2}\frac{\rho \left(r,\xi \right)}{r}dr=-N\xi$$

● inter-electronic interaction (see also Appendix)

(31c) $$\begin{array}{l} V_{\mathrm{ee}}\left[\xi \right]=\frac{N-1}{2N}\iint \frac{\rho \left(1\right)\rho \left(2\right)}{r_{12}}dv\left(1\right)dv\left(2\right)\\ \;=\left(N^{2}-N\right)\frac{5}{16}\xi \end{array}$$

With these results, the optimum atomic parameter is quantified by the electronic number as follows:

(32) $$\xi =\frac{21-5N}{16}\;$$

which immediately releases the working electronic density

(33) $$\rho^{0}\left(r,\xi \right)=\frac{N}{\pi }{\left(\frac{21-5N}{16}\right)}^{3}\exp\left[-\frac{21-5N}{16}\;2r\right]$$

Having the completely analytical density in terms of number of reactive electrons as in eq. (33), worth pointing here on the so called sign problem relating with its variation, e.g., its gradient, the gradient of its square root, etc. Although this problem usually arises in density functional theory when specific energy functionals are considered in gradient forms, see for instance ref. [49], there is quite instructive discussing the present behavior and its consequences.

For instance, one can adapt either eqs. (25b) or (25c) through considering the present form (32) for the orbital exponent to be

(25d) $$\frac{r}{n}=\frac{16}{21-5N_{\mathrm{bonding}}}$$

**Figure 2:**
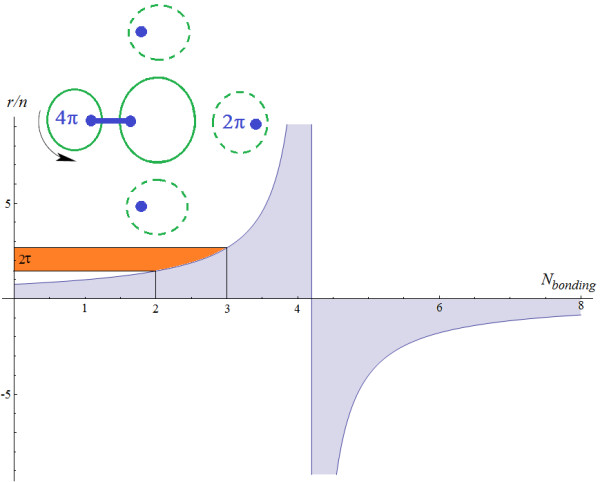
**Representation of the bonding length as a function of bonding electrons from valence atoms in molecule(s), based on eq. (25d), while marking the double golden ratio 2*τ *gap between the bonding lengths of the second and third bonding order, as well as the forbidden chemical bonding region for *N***_***bonding***_** ≥21/5 for the electrons participating in the same bonding.** Further connection of chemical bonding and the 4D space to model it is suggested by the inset picture illustrating the 2-fold (4*π*) spinning symmetry of the adduct atom respecting the bonding direction, after [51].

Here, one combines the frontier and maximum atomic radii with atoms-in-molecules phenomenology, as above indicated, to arrive to the present identification for the number of valence electrons possible to be involved in the same chemical bonding state as being *N*_*bonding*_ in (25d). Accordingly, the Figure 2 reveals interesting features of the present Slater-Parr-Bartolotti atomic density with quantum potential:

● the fact that the (covalent) bond length is proportional to the atomic radii and in inverse correlation with bonding order is well known [50], and this it is also nicely reflected in eq. (25d); however, changing the sign to negative radii as surpassing the threshold 21/5 and fixing in fact the limit *N*_*bonding*_=4, is consistent with maximum bond order met in Nature; it is also not surprising this self-released limit connects with golden ratio by the golden-spiral optimization of bond-order [51]; more subtle, it connects also with the 4*π* symmetry of two spherical valence atoms making a chemical bond (Figure 2, inset): such “spinning” reminds of the graviton symmetry [52] (the highest spherical symmetry in Nature, with spin equal 2) and justifies the recent treatments of chemical bonding by means of the quasi-particles known as bondons [10,53], as well as the use of the 4D complex projective geometry in modeling the chemical space as a non-Euclidian one, eventually with a time-space metrics including specific “gravitational effects” describing the bonding [51];

● the “gap” between the atomic systems contributing 2 to 3 electrons to produce chemical bond is about double of the golden ratio, ${\left(r/n\right)}_{N_{\mathrm{bonding}}=3}-{\left(r/n\right)}_{N_{\mathrm{bonding}}=2}≅2\tau$ ; therefore, this gap marks the passage from the space occupied by a pair of electrons and that required when the third electron is added on the same bonding state: it means that the third electron practically needs one golden measure (*τ*) to (covalently) share with each of the existing pairing electrons, while increasing the bond order to the level of three; it is therefore a *space representation of the Pauli exclusion principle* itself, an idea also earlier found in relation with dimensionless representation of a diatomic bonding energy (2*τ*) at its equilibrium bonding distance *τ*[54]; when the fourth electron is coming into the previous system, in order the maximum fourth order of bonding to be reach the chemical bonding space is inflating about five times more, yet forbidding further forced incoming electrons into the same space of bonding state as the bonding radius becomes negative in sign.

Having revealed the chemical bonding information carried by the density (33) when considered for combined valence atoms-in-molecules, it is next employed on energetically describing the atomic reactivity as a propensity for allowing electronic exchanging and bonding. As such, it leaves the total quantum (Bohmian) energy in (17) with the compact form

(34a) $$\begin{array}{l} E_{Q1}\left(a.u.\right)=\frac{25}{512}N^{2}-\frac{105}{256}N+\frac{441}{512}\\ \;=\frac{1}{512}{\left(21-5N\right)}^{2} \end{array}$$

Note that the actual working total energy is not that obtained by replacing the density (33) in eqs. (31a)-(31c) and then in total energy (30) because here the double-variational procedure was considered; that is, the first optimization condition was considered as in eq. (29), and the resulting (optimum) density (33) was then employed in the quantum energy (17), which in turn was obtained by applying the variational eq. (12) to the perceived phase transition in the Bohm eikonal wave-function (5). To emphasize the accuracy of eq. (17) over that of (30) with density (33), when one considers the last case, eq. (30) yields the following non-quadratic form for energy:

(34b) $$\begin{array}{l} E_{1}(a.u.)=-\frac{25}{512}N^{3}+\frac{105}{256}N^{2}-\frac{441}{512}N\\ \;=-\frac{N}{512}{\left(21-5N\right)}^{2} \end{array}$$

which is not appropriate for describing the valence state of an atom, as eq. (2) prescribes, despite being similar in form to the Bohmian-based result of eq. (34a). Thus, the previous limitation of the Parr-Bartolotti conclusion [33] and the paradox raised in describing the valence (parabolically) state with the optimized atomic density (33) are here solved by the double (or the orthogonal) variational implementation, as recently proved to be customary for chemical spaces [32]. In the light of this remark one may explain also the sign difference between the “physical” energy (34b) and that obtained for the “chemical” situation (34a): through simple variational procedure for “physical” energy (30) the result (34b) is inherently negative – modeling systems stability in agreement with the upper branch of eq. (22), whereas the double variational algorithm employing optimized density (33) into the Bohmian shaped energy (17) it produces the positive output (34a) associated with activation energy characteristic for chemical reactivity corresponding to the lower branch of eq. (22).

Therefore, to be accurate, one should consider the quantum potential related optimized energy (34a) instead of simply the orbital optimized one of eq. (34b). Therefore, assuming that eq. (34a) appropriately describes the atomic valence state in DFT (see the upper/reactive curve in Figure 1b), the next task is to search for the quantum valence charge for which the valence energy approaches its optimum value (or the “ground state” of the atomic chemical-reactivity, i.e. the previously golden-ratio quantification of the valence atomic state); to this aim, at this point, one can employ the golden ratio relationship (21a) and first rewrite eq. (34a) as

(35) $$E_{Q1}\left(a.u.\right)=\frac{5^{2}}{512}{\left(6.80\tau -N\right)}^{2}$$

which is minimized at the value

(36) N=6.80τ

However, one must again apply the double-variational procedure, now in terms of number of electrons, i.e., reconsidering eq. (36) with the golden ratio at the reactive (chemical) electronic level of eq. (22) such that a second equation is formed

(37) $$N=6.80\frac{Z}{N}$$

with the positive solution

(38) $$N_{\mathrm{REACT}}\equiv N^{**}=2.60768\sqrt{Z}$$

This expression avails of the significance of the maximum number of electrons, for a given atom, possibly engaged in a reactive environment by either (or both) accepting or (and) ceding electrons to or from its valence state, see Table 1.

**Table 1:** Synopsis of the critical charges in the physical ground state (N^*^) as well as for chemical reactive (valence) state (N^**^) for atoms of the first four periods of the periodic table of elements, as computed from the minimum point of associated interpolations of ionization and electronic affinities[33]**and of eq. (38), respectively**

**Atom**	**Z**	**I[eV]**	**A[eV]**	N^*^	N^**^
**H**	1	13.595	0.7542	0.558735	0.607681
**Li**	3	5.390	0.620	0.629979	0.516636
**B**	5	8.296	0.278	0.534672	0.830952
**C**	6	11.256	1.268	0.626952	0.387488
**O**	8	13.614	1.462	0.620309	0.375636
**F**	9	17.42	3.399	0.742422	0.823043
**Na**	11	5.138	0.546	0.618902	0.648699
**Al**	13	5.984	0.442	0.579755	0.402127
**Si**	14	8.149	1.385	0.70476	0.757049
**P**	15	10.484	0.7464	0.576651	0.0995049
**S**	16	10.357	2.0772	0.750876	0.430724
**Cl**	17	13.01	3.615	0.884779	0.751744
**K**	19	4.339	0.5012	0.630596	0.366618
**V**	23	6.74	0.526	0.584648	0.505999
**Cr**	24	6.763	0.667	0.609416	0.774976
**Fe**	26	7.90	0.164	0.5212	0.296616
**Co**	27	7.86	0.662	0.59197	0.549908
**Ni**	28	7.633	1.157	0.67866	0.798551
**Cu**	29	7.724	1.226	0.688673	0.0427917
**Se**	34	9.75	2.0206	0.761417	0.205262
**Br**	35	11.84	3.364	0.896885	0.427249
**Rb**	37	4.176	0.4860	0.631707	0.861904
**Zr**	40	6.84	0.427	0.566584	0.492423
**Nb**	41	6.88	0.894	0.649348	0.697305
**Mo**	42	7.10	0.747	0.617582	0.899704
**Rh**	45	7.46	1.138	0.680006	0.492856
**Pd**	46	8.33	0.558	0.571796	0.686153
**Ag**	47	7.574	1.303	0.707782	0.87736
**Sn**	50	7.342	1.25	0.705187	0.439089
**Sb**	51	8.639	1.05	0.638358	0.622567
**Te**	52	9.01	1.9708	0.779975	0.804255
**I**	53	10.454	3.061	0.91404	0.984204

**Figure 3:**
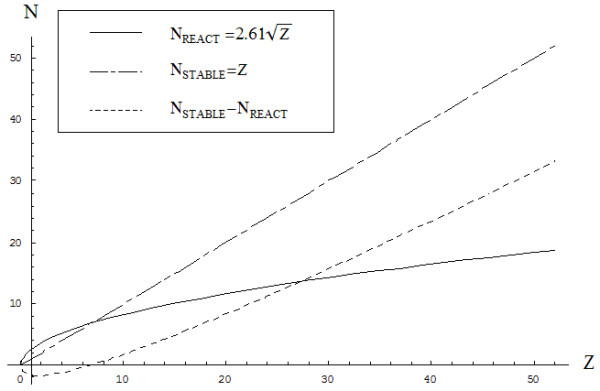
**The comparative shapes of the valence electrons to be engaged in chemical reactivity (continuous curve) computed using eq. (38) based on the combined optimal Bohm total energy (35) with the golden ratio imbalance of eq. (22), respecting the stable physical case (dot-dashed curve), and of their differences (dashed curve); all originate at the 0**^**th**^**atom (the neutron, Z=0).**

The result of this process is different from the expected physical result (*N*_*STABIL*_*=Z*) according to the upper branch of eq. (22), which is higher than the physical one until reaching the carbon system (*Z*_*INTRCHANGE*_=6.8), while continuing below it thereafter (see Figure 3).

**Figure 4:**
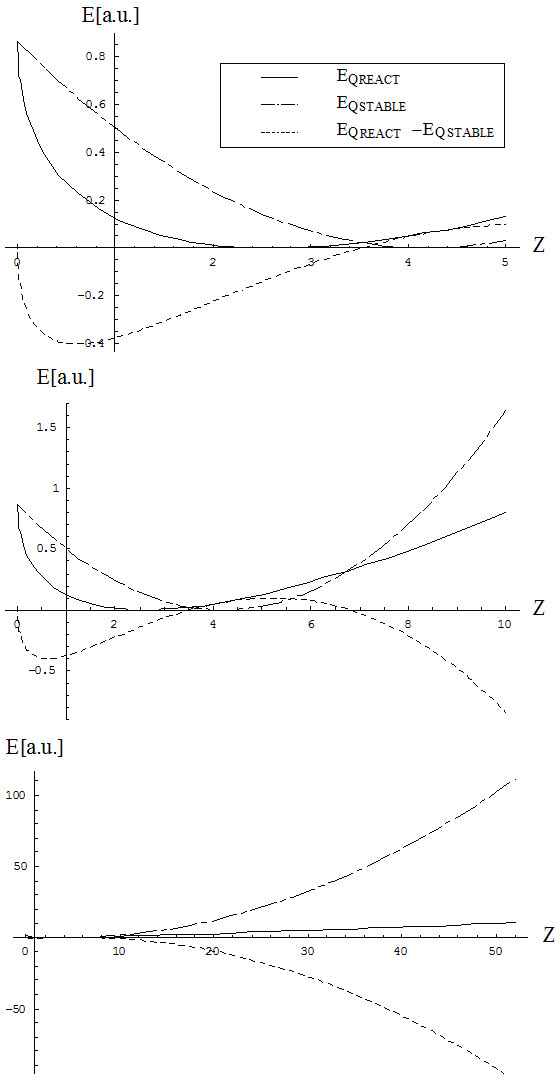
**The same comparative shapes shown in Figure**3**, here at the level of energy (34a) specialized for the reactive and the stable N(Z) dependencies of Figure**3**; the various plots successively display increasingly large atomic Z-ranges to better emphasize the chemical vs. physical behavior (see text).**

The above interchange (effective) atomic number through which the chemical (reactive) state is associated with lower charge respecting the physical state may be also be found at the energetic level based on quantum equation (34a), as specialized for the two branches of Figure 3 for the N(Z) dependence. Thus, the chemical (reactive) state takes the analytic form

(39) $$\begin{array}{l} E_{Q1}(N_{\mathrm{REACT}}\to 2.60768\sqrt{Z})\\ \;=0.861328-1.06956\sqrt{Z}+0.332031\;Z \end{array}$$

and interchanges with the ground state *E*_*Q*1_(*N*_*STABLE*_ → *Z*) at the points {3.5,6.8}, as observed also from Figure 4; however, the interchanging point beyond which all chemical atomic systems are more stable in the chemical or reactive state than in the physical ground state is consistently recovered.

Nevertheless, the energetic analysis also reveals the atomic systems Be, B and C to be situated over the corresponding physical stable states; this may explain why boron and carbon present special chemical phenomenology (e.g., triple electronic bonds and nanosystems with long C-bindings, respectively), which is not entirely explained by ordinary physical atomic paradigms [55-60].

The energetic discourse may be complete with the electronegativity and chemical hardness evaluations by applying the DFT definitions (3) and (4) to physical and chemical energies, respectively. In the first case, expression (34b) is applied to provide the following so-called “universal” forms of Parr and Bartolotti [33]:

(40) $$\chi_{PB1}=-{\left(\frac{\partial E_{1}}{\partial N}\right)}_{N=1}=\frac{3}{16}\left(a.u.\right)=5.1\left(eV\right)$$

(41) $$\eta_{PB1}=\frac{1}{2}{\left(\frac{\partial^{2}E_{1}}{\partial N^{2}}\right)}_{N=1}=\frac{135}{512}\left(a.u.\right)=7.17\left(eV\right)$$

The result, nevertheless, appears to be an unusually higher increase in chemical hardness than in electronegativity, which certainly cannot be used to model a reactive-engaged tendency because it is more stable (by chemical hardness) than reactive (by electronegativity); it is, however, consistent with the physical stability of the system, provided by the single variational procedure through which eq. (34b) was produced.

Instead, to chemically model reactivity, the double variation procedure is applied and eq. (34a) is substituted into eqs. (3) and (4), though by considering also the double reactive procedure for charge as well, i.e., by considering eq. (38) with the golden ratio information of (22) to respectively yield the results

(42) $$\begin{array}{l} \chi_{PB2}=-{\left({\left({\left(\frac{\partial E_{Q1}}{\partial N}\right)}_{N=2.60768\sqrt{Z}}\right)}_{Z=\tau N}\right)}_{N=1}\\ \;=0.209963(a.u.)=5.713(eV) \end{array}$$

(43) $$\eta_{PB2}=\frac{1}{2}\left(\frac{\partial^{2}E_{Q1}}{\partial N^{2}}\right)=\frac{25}{512}\left(a.u.\right)=1.3286\left(eV\right)$$

Remarkably, the actual electronegativity of (42) obtained by the quantum Bohm and golden ratio double procedure yields sensible results similar to those of the single variational approach (40); however, the chemical hardness of (43) is approximately 5-fold lower than its “stable” counterpart (41), affirming therefore the manifestly reactive framework it produces – one described by a quadratic equation (34a) instead of a cubic one (34b).

### Charge waves in gauge chemical reactivity

Finally, one considers the chemical reactivity discussion as based on the gauge reaction that equilibrates the chemical bond by symmetrical bond polarities [25]

(44a) $$A^{-}+B^{+}=A-B=A^{+}+B^{-}$$

such that the reactive electrons are varied on the reunited intervals of eq. (1); such analysis was previously employed to fundament systematic electronegativity and chemical hardness definitions by the averaging (through the integration) factor

(44b) $$0.5=\frac{1}{\int_{-1}^{+1}dN}$$

along the reaction path accounting for the acidic (electron accepting, 0 ≤ *N* ≤ +1) and basic (electron donating, –1 ≤ *N* ≤ 0) chemical behaviors.

**Figure 5:**
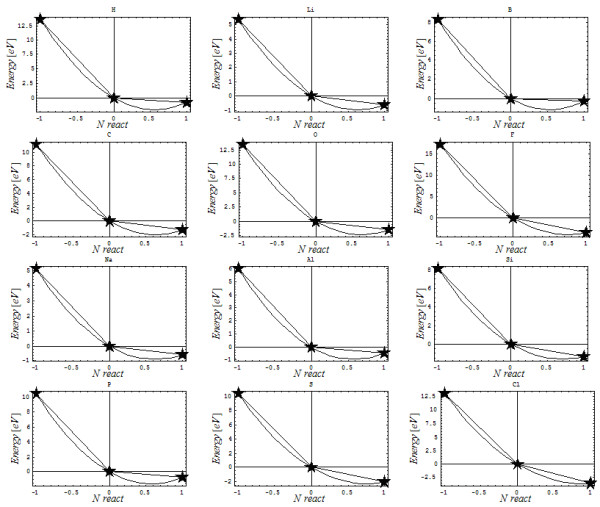
**A graphical interpolation for selected elements of Table**1**in terms of their ionization, neutral and affinity states, aiming to determine the critical (displaced) charge of the DFT ground state, as prescribed by Figure**1b.

In this scaled (gauge) context of reactivity, the foregoing discussion is dedicated to investigating the link between the critical ground state charge (N^*^) and the valence or reactive state (N^**^). While the first appears as a consequence of naturally fitting the three points in Figure 1 (the ionization, neutral and affinity states), with the effect of biasing the minimum of the energetic curve in Figure 1b with respect to the apparent Parr-DFT curve in Figure 1a, and is thus derived graphically (see Figure 5), the valence charge is based on the combined quantum energy and golden ratio information in eq. (38). Both are reported for the indicated number of atomic systems of the periodic table of elements in Table 1. One notes, for instance, that while the critical ground state charge N^*^ always lies in the range [0.5,1], the valence charge N^**^ may span the interval [0,1]; one may interpret such behavior as being associated with the difference between the fraction Â½ and integer “1” in driving the principles of chemical reactivity and the electrophilicity equalization principle in particular, when the “quantum transition” 1/2→1 is required in the energy exchange of chemical systems for it to be valid for both electronegativity and chemical reactivity principles [61]; nevertheless such scaling it is equivalent with above acidic-basic gauge averaging of eq. (44b). This way, the valence charge problem may be extended to the interval [0,2], at its turn seen as a gauge transformation of the chemical reactivity charge domain [−1, +1], where one reencounters the challenging problem of whether the “One electron is less than half what an electron pair is” [62], the response to which is generally complex but may here be approached through the following steps.

**Figure 6:**
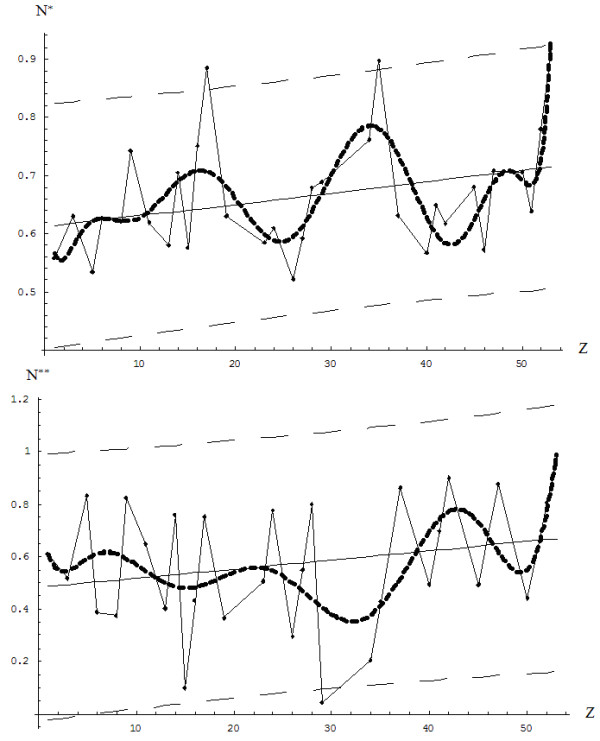
**The critical ground state and valence charge points for the elements of Table**1**and their 10**^**th**^**-order continuous interpolations according to eqs. (45a) and (45b).**

First, by employing the data presented in Table 1, one constructs the so-called “continuous” ground and valence charge states by appropriately fitting over the first four periods of elements, here restrained to 10^th^-order polynomials. This is performed by interpolating every three points of the 32 elements presented in Table 1, although by spanning the atomic number range *Z* ∈ [1, 53], thus yielding (see also the allied representations of Figure 6):

(45a) $$\begin{array}{l} NC^{*}=0.677771-0.193006Z+0.104303Z^{2}\\ \;-0.0242757Z^{3}+0.00302359Z^{4}\\ \;-0.000220204Z^{5}+9.79371\cdot 10^{-6}Z^{6}\\ \;-2.6894\cdot 10^{-7}Z^{7}+4.4448\cdot 10^{-9}Z^{8}\\ \;-4.05002\cdot 10^{-11}Z^{9}+1.56254\cdot 10^{-13}Z^{10} \end{array}$$

(45b) $$\begin{array}{l} NC^{**}=0.768074-0.224502Z+0.076654Z^{2}\\ \;-0.0107337Z^{3}+0.000667575Z^{4}\\ \;-0.000012746Z^{5}-6.0246\cdot 10^{-7}Z^{6}\\ \;+4.01411\cdot 10^{-8}Z^{7}-9.49568\cdot 10^{-10}Z^{8}\\ \;+1.05449\cdot 10^{-11}Z^{9}-4.58533\cdot 10^{-14}Z^{10} \end{array}$$

Equations (45a) and (45b) are then combined into a sort of special charge wave function based on their difference on the golden ratio scale (see Figure 6 for graphical representation)

(46) $$\Psi_{Z}=\tau \left(NC_{Z}^{*}-NC_{Z}^{**}\right)$$

with the peculiar property that its square-integrated form over the Z-range of interpolation gives

(47) $$\int_{1}^{53}{\left|\Psi_{Z}\right|}^{2}dZ=0.667233\approx \tau$$

The result (47) has the following conceptual fundamental quantitative interpretation: the difference between the ground and valence optimum charges is regulated by the golden ratio scale, or in other terms,

(48) $$\int_{1}^{53}{\left(NC_{Z}^{*}-NC_{Z}^{**}\right)}^{2}dZ\approx \frac{1}{\tau }=1+\tau$$

such that it provides a sort of normalization corrected by the golden ratio value; it also fulfills the interesting relationship:

(49) $$\sqrt{\int_{1}^{53}{\left(NC_{Z}^{*}-NC_{Z}^{**}\right)}^{2}dZ}\approx 2\tau$$

In any case, the present analysis provides the qualitative result that the difference between the critical ground state and optimal valence charges is more than half of an electronic pair, giving rise to the significant notion that chemical reactivity is not necessarily governed by a pair of electrons but governed by no less than half of a pair and is related to the golden ratio (*τ* > 0.5).

**Figure 7:**
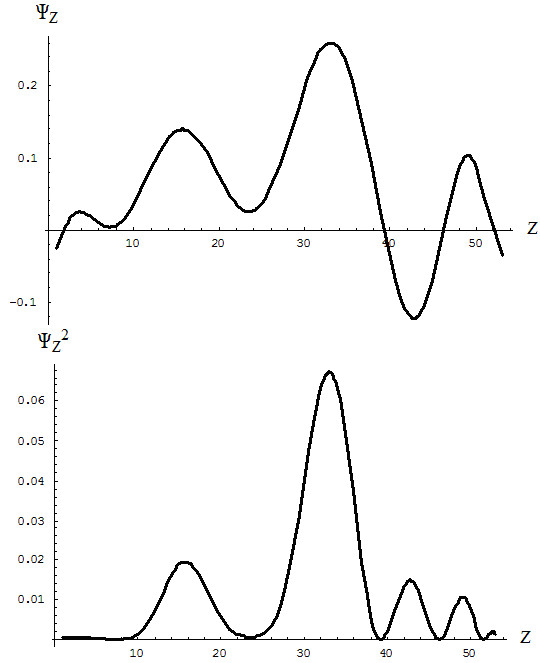
**The linear and quadratic charge “wave function” of eq. (46).**

However, fractional values in general and those related to the golden ratio particular, may be interpreted as a consistent manifestation of the quantum mechanical (i.e., wave functional) approach of chemical phenomena, here at the reactivity level. Moreover, the quadratic critical charge function (46), as shown in Figure 7, clearly reveals that a higher contribution to electronic pair chemistry is given by the third period of elements and by the third and fourth transitional elements in particular, a result that nicely agrees with the geometrical interpretation of the chemical bond, particularly the crystal ligand field paradigm of inorganic chemistry [9].

Also a local analysis of the type of charge that is dominant in atomic stability, i.e., the critical physical ground state or the chemical valence reactive state based on eqs. (45a) and (45b), respectively, may be of considerable utility in refined inorganic chemistry structure-reactivity analysis. To the same extent, it depends on the degree of the polynomials used to interpolate the critical and valence charges over the concerned systems; however, through the present endeavor, we may assert that the analysis should be of the type (48), which in turn remains a sort of integral version of the *imbalance equation* (20a), in this case for the ground-valence charge gap states of a chemical system.

## Conclusions

Aiming to hint at the solution to the current debate regarding the physical vs. chemical definition of an atom and as a special stage of a larger project regarding quantum chemical orthogonal spaces, the present work addresses the challenging problem of defining and characterizing valence states with respect to the ground state within conceptual density functional theory. We are aware of the earlier warnings raised by Parr and Bartolotti and others [18,33,63] regarding the limits of density functional theory and of the total energy of atomic systems combined with a Slater-based working density to provide a quadratic form in terms of system charge, as required by the general theory of chemical reactivity of atoms and molecules in terms of electronegativity and chemical hardness. Fortunately, we discovered that the Bohmian form of the total energy of such atomic systems provides, instead, the correct behavior, although it is only density-function-dependent and not a functional expression. Moreover, this finding was reached through the so-called double variational procedure, which, as emphasized earlier, was likely to reproduce the chemical reactivity principles of electronegativity and chemical hardness in an analytical manner; however, such a double analytical variational approach is consistent with the recent advanced chemical orthogonal spaces approaches of chemical phenomenology [64] as being at least complementary to the physical description of many-electronic systems when they are engaging in reactivity or equilibrium as the atoms-in-molecules Bader theory prescribes [9,42]. With the present Bohmian approach, the total energy is in fact identified with the quantum potential, thus inherently possessing non-locality and appropriate reactivity features, which are manifested even over long distances [10,11,53]; this also generalizes the previous Boeyens electronegativity formulation of electronegativity [5,6] from the direct relationship between a quantum potential and its charge derivative. The double algorithm was also implemented to discriminate the valence from the ground state charges, this time by using the golden ratio imbalance equation as provided by adaption of the Heisenberg type relationship to chemical reactivity for atoms. This corresponds to an analytical unfolding of the physical and chemical imbalance of the electronic charge stability of atomic systems, paralleling the deviation from the equal electron-to-proton occupancy in physical systems toward electron deficiency in the valence states of chemical systems. This dichotomy was implemented by the golden ratio presented in eq. (22). As a consequence, the difference between valence and ground state charge systems is naturally revealed and allows for the explanation of chemical reactivity and bonding in terms of fractional electron pairs, althrough driven by the golden ratio under the so-called physical-to-chemical charge difference wave function and associated normalizations, all of which represent elaborated or integral forms of the basic imbalance atomic equation. The present results are based on 10^th^-order polynomial fitted over 32 elements from the first 54 elements of the first four periods of periodic table of elements and can be further pursued by performing such systematic interpolations that preserve the golden ratio relationships, as advanced herein; they may also provide a comprehensive picture of how valence electrons may always be projected/equalized/transposed into ground state electrons within the perspective of further modeling chemical reactions when chemical reactivity negotiates the physical molecular stabilization of atoms in molecules.

## Endnotes

^a^ For circular orbits, the lowest ones in each atomic shells – including the valence ones, one has Δ*O*=Δ*r*=2*πr*, with *r* the orbital radii thereof, while *O=p* is the fixed particle’s momentum on that orbit; therefore, when combined into eq. (18b) they provide the celebrated Bohr-de Broglie relationship *rp=nħ* solving the atomic spectra of Hydrogen atom in principal quantum numbers (*n*).

## Appendix: Semi-classical inter-electronic energy

**Figure 8:**
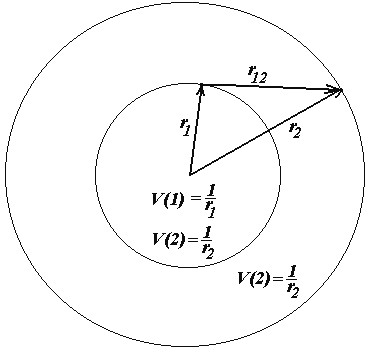
**Representation of the space regions of the 1**^**st **^**and 2**^**nd **^**electrons, their potential influences and reciprocal interaction **[21,48]

For the inter-electronic interaction, see Figure 8; in evaluating *V*_*ee*_[*ξ*] of eq. (31c), the two-electronic density is approximated by the Coulombic two mono-electronic density product, thus neglecting the second-order density matrix effects associated with the exchange-correlation density.

However, for the analytical evaluation of the electron–electron repulsion energy using the density (23b), much care must be taken. For instance, one has to use the electrostatic Gauss theorem, which states that the classical electrostatic potential outside a uniform spherical shell of charge is just what it would be if that charge were localized at the center of the shell and that the potential everywhere inside such a shell is that at the surface, [21,48] see Figure 8. Therefore, the electronic repulsion energy becomes

(A1) $$\begin{array}{l} V_{\mathrm{ee}}[\xi ]=\frac{N-1}{2N}\iint \frac{\rho \left(1\right)\rho \left(2\right)}{r_{12}}dv\left(1\right)dv\left(2\right)\\ \;=\frac{N-1}{2N}\int_{0}^{\infty }4\pi \;r_{1}^{2}N\frac{\xi^{3}}{\pi }\exp(-2\;\xi \;r_{1})dr_{1}\\ \;x\;\left[\int \frac{\rho \left(2\right)}{r_{12}}dv\left(2\right)\right]\\ \;=\frac{N-1}{2N}\int_{0}^{\infty }4\;r_{1}^{2}N\xi^{3}\exp(-2\;\xi \;r_{1})dr_{1}\\ \;x\;\{4\pi \;N\frac{\xi^{3}}{\pi }[\int_{0}^{r_{1}}\frac{r_{2}^{2}\exp\left(-2\xi \;r_{2}\right)}{r_{1}}dr_{2}\\ \;+\;\int_{r_{1}}^{\infty }\frac{r_{2}^{2}\exp\left(-2\xi \;r_{2}\right)}{r_{2}}dr_{2}]\;\}\\ \;=\frac{N-1}{2N}N^{2}16\xi^{6}\int_{0}^{\infty }r_{1}^{2}\exp(-2\;\xi \;r_{1})dr_{1}\\ \;x\;\{\;[\int_{r_{2}\to r_{1}}^{r_{2}\to \infty }\left(\frac{1}{r_{1}}\equiv \frac{1}{r_{2}}\right)r_{2}^{2}\exp\left(-2\xi \;r_{2}\right)dr_{2}\\ \;+\;\int_{r_{1}}^{\infty }r_{2}\exp\left(-2\xi \;r_{2}\right)dr_{2}\}]\\ \;=\frac{N-1}{2N}N^{2}32\xi^{6}\int_{0}^{\infty }r_{1}^{2}\exp(-2\;\xi \;r_{1})dr_{1}\\ \;x\;\left[\int_{r_{1}}^{\infty }r_{2}\exp\left(-2\xi \;r_{2}\right)dr_{2}\right]\\ \;=\frac{N-1}{2N}N^{2}\frac{32\xi^{6}}{{\left(2\xi \right)}^{5}}\int_{0}^{\infty }{\left(2\xi \;r_{1}\right)}^{2}\exp(-2\;\xi \;r_{1})d(2\xi \;r_{1})\\ \;x\;\left[\int_{2\xi \;r_{1}}^{\infty }2\xi \;r_{2}\exp\left(-2\xi \;r_{2}\right)d\left(2\xi \;r_{2}\right)\right]\\ \;\equiv \frac{N-1}{2N}N^{2}\xi \;\int_{0}^{\infty }s^{2}\exp(-s)\left[\int_{s}^{\infty }t\exp\left(-t\right)dt\right]ds\\ \;=\frac{N-1}{2N}N^{2}\xi \int_{0}^{\infty }s^{2}\exp(-s)\left[\left(1+s\right)\exp\left(-s\right)\right]ds\\ \;=\frac{N-1}{2N}N^{2}\xi \left(\frac{2!}{2^{3}}+\frac{3!}{2^{4}}\right) \end{array}$$

which recovers the expression presented by eq. (31c), when the Slater integral type of Eq. (28) is also employed. Note that the electron–electron repulsion term was written by also considering the Fermi-Amaldi (*N*-1)/*N* factor [13], which ensures the correct self-interaction behavior: when only one electron is considered, the self-interaction energy must be zero, *V*_*ee*_ (*N*→1)→0.

## Competing interests

The author declares he has no competing interests.
